# Differential Conserted Activity Induced Regulation of Nogo Receptors (1–3), LOTUS and Nogo mRNA in Mouse Brain

**DOI:** 10.1371/journal.pone.0060892

**Published:** 2013-04-11

**Authors:** Tobias E. Karlsson, Josefin Koczy, Stefan Brené, Lars Olson, Anna Josephson

**Affiliations:** 1 Department of Neuroscience, Karolinska Institutet, Stockholm, Sweden; 2 Department of Neurobiology, Caring Sciences and Society, Karolinska Institutet, Stockholm, Sweden; University of Fukui, Japan

## Abstract

Nogo Receptor 1 (NgR1) mRNA is downregulated in hippocampal and cortical regions by increased neuronal activity such as a kainic acid challenge or by exposing rats to running wheels. Plastic changes in cerebral cortex in response to loss of specific sensory inputs caused by spinal cord injury are also associated with downregulation of NgR1 mRNA. Here we investigate the possible regulation by neuronal activity of the homologous receptors NgR2 and NgR3 as well as the endogenous NgR1 antagonist LOTUS and the ligand Nogo. The investigated genes respond to kainic acid by gene-specific, concerted alterations of transcript levels, suggesting a role in the regulation of synaptic plasticity, Downregulation of NgR1, coupled to upregulation of the NgR1 antagonist LOTUS, paired with upregulation of NgR2 and 3 in the dentate gyrus suggest a temporary decrease of Nogo/OMgp sensitivity while CSPG and MAG sensitivity could remain. It is suggested that these activity-synchronized temporary alterations may serve to allow structural alterations at the level of local synaptic circuitry in gray matter, while maintaining white matter pathways and that subsequent upregulation of Nogo-A and NgR1 transcript levels signals the end of such a temporarily opened window of plasticity.

## Introduction

Structural synaptic plasticity in the central nervous system is under tight control, and partly mediated by the Nogo signaling system [1], [2], [3]. In 1988 it was shown that the inihibitory effects of myelin could be counteracted by antibodies that bound to 35 and 250 kd inhibitory proteins [4], [5]. The inhibitory protein was cloned and named Nogo [6], [7], [8], and it was shown that Nogo had three different isoforms, A, B and C. The first receptor to be found for Nogo was the Nogo-66 receptor (NgR1) [9]. Being GPI-linked and lacking an intracellular domain, NgR1 requires co-receptors in order to transduce signals to the cytosol. Identified co-receptors include p75 [10], Troy [11], [12] and Lingo-1 [13] and through combinations of these co-receptors Nogo-NgR1 signaling can result in activation of RhoA [14]. Paired immunoglobulin-like receptor B (PirB) is another receptor for Nogo, and two additional ligands, MAG [15], and OMgp [16], [17] can also bind to both NgR1 [17], [18] and PirB [15]. The most well studied form of Nogo is Nogo-A, and although it was first identified in myelin [4], [5], subsequent studies found that it was also expressed by neurons [19], [20], suggesting functions for the Nogo-system not related to myelin. NgR1 is expressed by neurons and downregulated in several different situations associated with increased neuronal activity and plasticity [21], [22], [23]. Strong support for an important role of NgR1 in synaptic plasticity in the uninjured nervous system came when it was shown that mice that lack NgR1 retain plasticity in their ocular columns even after the normal developmental closure of such plasticity [24]. To investigate if the downregulation of NgR1 is of importance for the formation of lasting memories we created a mouse with inducible overexpression of NgR1 in forebrain neurons and found that while “short” term memory (day to day) was unaffected, the ability to form memories lasting longer than 30 days was severely impaired [25] when the transgene was activated. NgR1 has two homologs, NgR2 and NgR3 [26], with partly overlapping, partly specific brain expression profiles. NgRs 1–3 are all expressed in cerebral cortex and hippocampus, while NgR1 and NgR2, but not NgR3 are expressed in thalamus. A further difference is that NgR2 shows marked expression in striatum, while this is not the case for NgR1 and NgR3. MAG has been found to bind to NgR2 but not to NgR3; neither Nogo-A nor OMgp has been reported to bind to these homologous receptors [27]. Recently,it was shown that both NgR1 and NgR3 could bind to chondroitin sulfate proteoglycans [28], thereby identifing the first ligand for NgR3 and increasing the number of ligands that can bind to NgR1. In the search for molecules that would affect the growth of the lateral olfactory tract, a protein named LOTUS was found and shown to be an endogenous antagonist of NgR1, able to compete with Nogo-A for NgR1 [29].

To increase understanding of the possible role of the Nogo signaling system in plasticity, we now provide a detailed spatial and temporal description of neural activity-induced alterations of the NgR 1–3, Lotus and Nogo-A transcriptome in the brain, using quantitative in situ hybridization. Our findings suggest a concerted pattern of alterations of these five genes that together may allow locally restricted structural plasticity during a restricted time window.

## Methods

### Ethics Statement

The experiments were approved by the Stockholm North animal ethics committee (N580/11).

### Animals

Male mice (C57/Bl6) (around 12 weeks of age) were purchased from Charles-River (Sulzfeld, Germany) and allowed to habituate for >2 weeks. Mice were group housed with litter mates in cages with access to food and water *ad libitum.* They were kept on a 12/12 hour light/dark cycle; lights were on between 06∶00 and 18∶00. The temperature was kept at 22–23°C with a relative humidity of 60%. In the cages, mice had access to a small paper house and tissue paper as “enrichment”.

### Kainic Acid Administration

Kainic acid, 30 mg/kg i.p., (a dose that does not induce significant neuronal death in C57 mice [30]) was administrated to mice as a single dose and behavior was monitored for seizure activity using a standardized seizure scoring scale [31]. Only mice that received a grade IV (rearing) or V (falling over) seizure score were included in the subsequent analysis. Mice were sacrificed (by decapitation) at 2 (n = 7), 4 (n = 8), 12 (n = 7), 24 (n = 4) and 72 (n = 5) hours after the kainic acid injection. Brains from treated and non-treated mice (n = 6), “0” h, were sectioned (14 µm) using a cryostat (Microm). For the anlysis, 4 sections per animal were used (with an intervall of 10 sections).

### In situ Hybridization

We performed in situ hybridization as previously described [32]. To ensure that our selected probes were specific, they were first aligned to all publicly known sequences using the UCSC genome browser http://genome.ucsc.edu/) and folding energy was assed using Mfold (Version 3.2) [33], [34]. We then created at least two oligonucleotides that targeted different parts of the mRNA of interest. We analyzed the hybridization of both oligonucleotide probes before one was chosen for the experiments (expression patterns were always identical for probes directed at the same transcript, but some probes generated stronger signals and were chosen for subsequent experiments), expression patterns were also compared to previously published data when available.

The following ^33^P-labeled oligonucleotide DNA probes were used: NgR1: “GTG CAG CCA CAG GAT AGT GAG ATT TCG GCA TGA CTG GAA GCT CGC AGC TTC GGG GCG”, NgR2: “AGG GCG CTC AGT CCA CAC TTA TAG AGG TAG AGG GCG TGA AGC TTC”, NgR3: “AAG GAC AGC GGC ACT GAG GAG AAG TTG TTG GCC TGG CAG CTC ACG GT”, LOTUS: “ACA GAC AGT GGC TGA GCC ATG GAC TCT CCA TGT GAC AAG ATG AGA TAA AGC A”.

Hybridized and rinsed sections were air-dried and exposed to X-ray film (Biomax, Eastman Kodak) for 14–21 days. The films were developed and scanned using a high resolution scanner (v750 PRO, Epson). The digitized images were used to analyze optical density of regions of interest, using an image analysis program (ImageJ v. 1.32j, http://rsb.info.nih.gov/ij/). No change or adjustments were made to the scanned file prior to analysis. On the films a ^14^C step standard (Amersham) was also included to obtain a standard curve that was used to convert measured values to nCi/g. Measurements were performed on representative sections (4 per mouse) from each region studied, and a mean value calculated for each animal and normalized to the expression of mice not exposed to kainic acid. Data are expressed as mean ± SEM. For in situ images shown in figures, the contrast was adjusted (same exact setting for all images of a particular probe) and small dust particles and background were digitally removed. Original images are available from the authors.

### Statistics

Data were analyzed using a one-way ANOVA and when a significant between group effect was seen this was followed up with an appropriate post-hoc test (Dunnet’s or Games-Howell) using SPSS. No injection (t = 0) was always used as the reference group. In text, the significance presented will refer to the ANOVA between group effect if nothing else is stated.

## Results

### Kainic Acid-induced Increase of Neuronal Acitivity

High levels of neuronal activity were induced in mice by injecting kainic acid at a dose of 30 mg/kg (shown not to induce significant cell loss in c57/Bl6 mice [30]). The resuling seizure behavior was assessed and only mice with seizure scores of IV (rearing) or V (falling over) were used in the subsequent analysis. There was no significant difference in seizure scores between the groups that received kainic acid.

### NgR1 mRNA is Downregulated by Kaninic Acid

**Figure 1:**
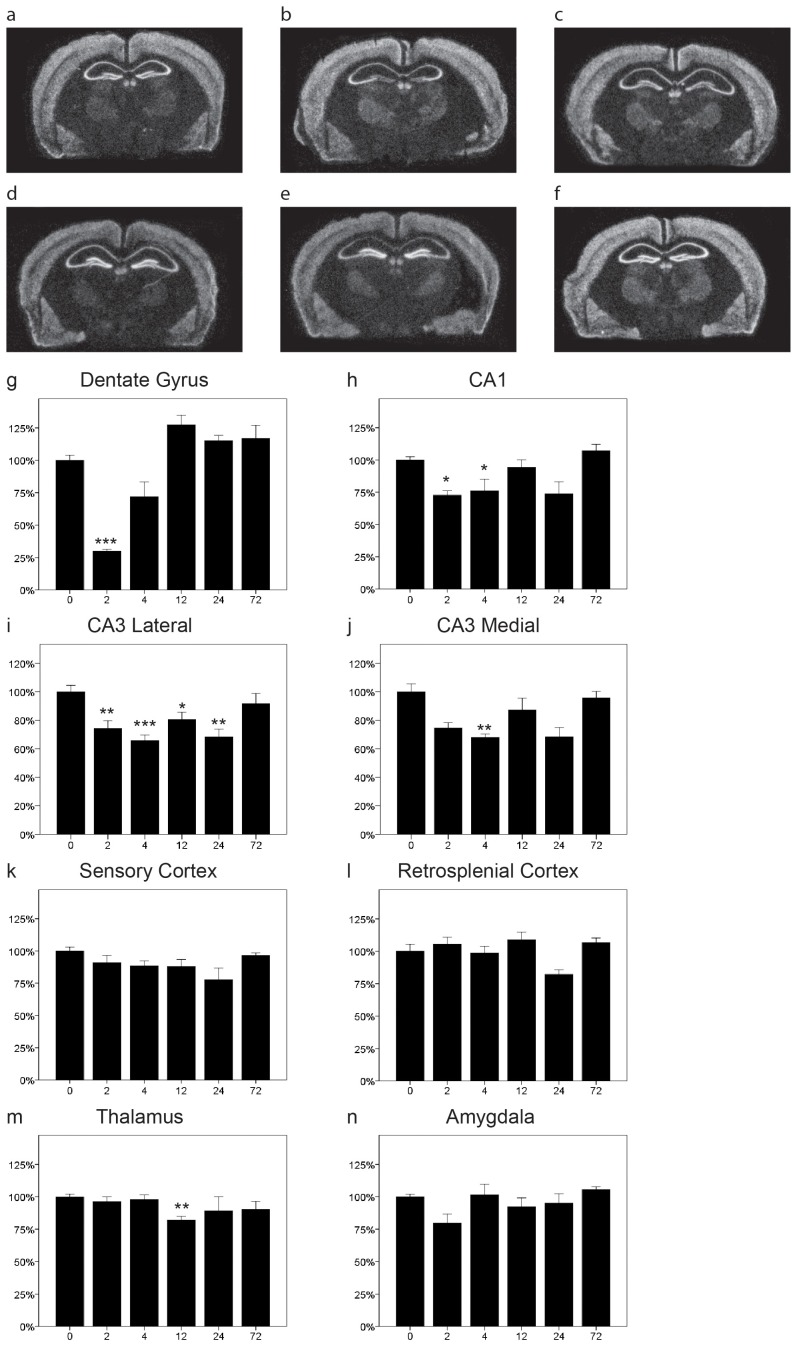
Distribution and regulation of NgR1 mRNA in the mouse brain. Representative coronal sections of control (a) and kainic acid injected (b 2 h, c 4 h, d 12 h, e 24 h, f 72 h) mice probed for NgR1 mRNA by in situ hybridization. Quantitation of NgR1 mRNA levels in eight brain areas (g–n). NgR1 levels decreased significantly after 2 h in the dentate gyrus (g) and in CA1 between 2–4 hours (h). In lateral CA3 NgR1 mRNA was significantly downregulated between 2 and 24 hours (i) and at 4 hours in medial CA3 (j). Levels did not change significantly in sensory or retrosplenial cortex (k,l). Expression levels in thalamus were quite stable but showed a significant reduction at 12 h (m). No significant change was detected in amygdala (n). Error bars represent S.E.M. and all expression values were normalized to expression in non-injected control mice (t = 0) for g–n. *p<0.05, **p<0.01, ***p<0.001 compared to 0 h.

We have previously shown in rats and mice that NgR1 is rapidly downregulated after kainic acid injections [22], [25]. Here we confirm the occurence of efficient downregulation of NgR1 mRNA in response to the excitotoxin kainic acid in mice. We now provide a more detailed temporal resolution of the decrease and recovery of NgR1 mRNA levels and extend the analysis to more regions (Fig. 1). NgR1 was downregulated in the dentate gyrus as have previously been shown (Fig. 1 g, P<0.001), CA1 (Fig. 1h, p = 0.003) and in both lateral (Fig. 1i, p<0.001) and medial (Fig. 1j, p = 0.001) CA3. Of the two cortical locations analyzed we found a significant change in retrosplenial cortex (Fig. 1 l, p = 0.047) but no significant change in sensory cortex (Fig. 1k, p = 0.11).The downregulation appeared to be somewhat faster and of a larger magnitude in the dentate gyrus than in CA1 and CA3. Taken together, there was a tendency for biphasic recovery and/or overshoot of NgR1 mRNA levels, with a higher mean NgR1 mRNA level at 12 hr than at either the 4 or 24 hr. The thalamic levels of NgR1 were rather stable but a significant downregulation was seen at 12h (Fig. 1 m, p = 0.014). In amygdala no significant change was detected (Fig. 1n, p = 0.096).

### NgR2 mRNA is Regionally Upregulated by Kainic Acid

**Figure 2:**
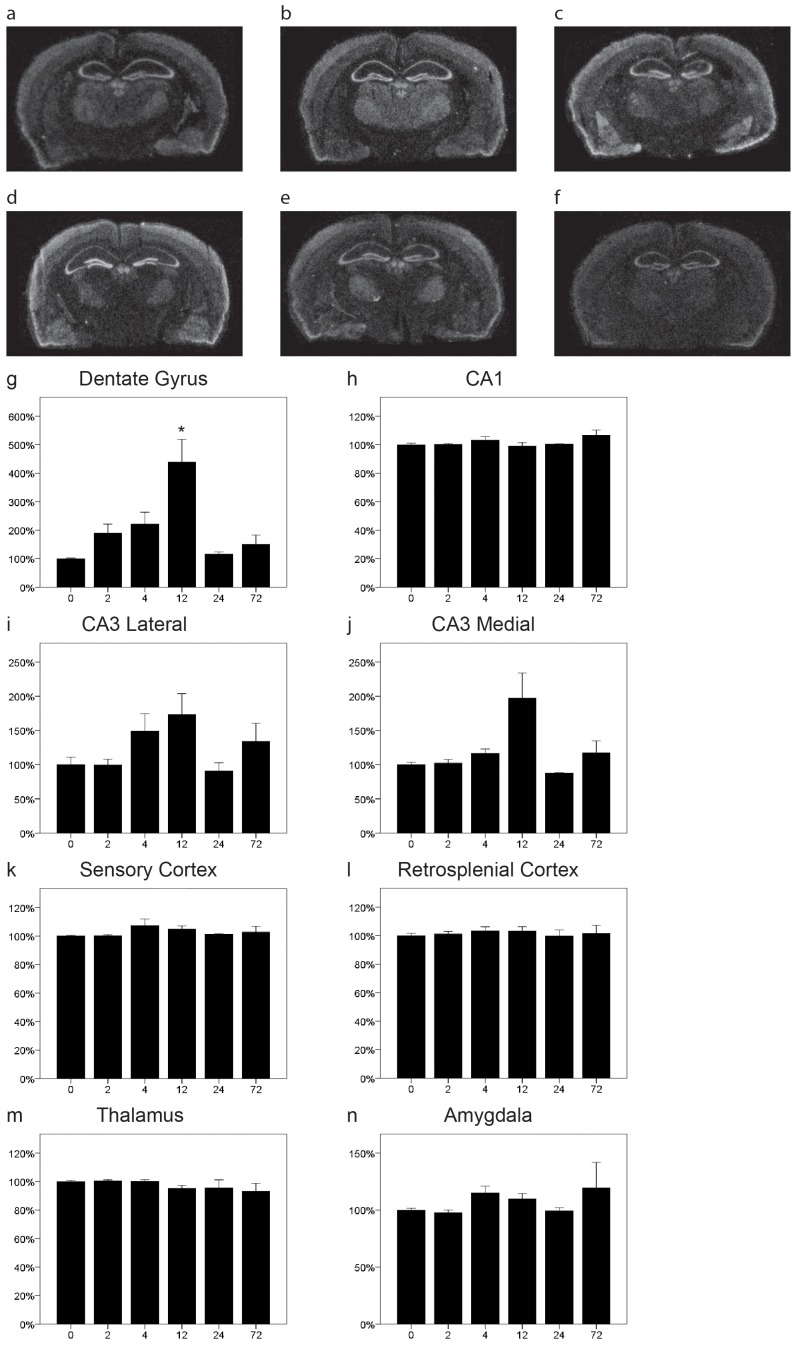
Distribution and regulation of NgR2 mRNA in the mouse brain. Representative coronal sections of control (a) and kainic acid injected (b 2 h, c 4 h, d 12 h, e 24 h, f 72 h) mice probed for NgR2 mRNA by in situ hybridization. Quantitation of NgR2 mRNA levels in eight brain areas (g–n). NgR2 mRNA levels were robustly increased in the dentate gyrus, being significant at 12 h (g). In CA1 the expression was stable and not significantly altered at any time point (h). There was a significant overall effect of kainic acid on NgR2 mRNA expression in medial (j) but not lateral CA3 (i), but no specific time point was significantly different from controls. The expression levels were very stable in sensory (k) and retrosplenial cortex (l) as well as in thalamus (m) and amygdala (n). Error bars represent S.E.M. and all expression values were normalized to expression in control mice (t = 0). *p<0.05, **p<0.01 compared to 0 h.

There was a widespread expression of NgR2 mRNA in many regions of the brain (Fig. 2). Expression was ubiquitous in the cerebral cortex with higher levels in the outer than in the inner cortical layers, and there was a distinct expression in the hippocampal formation, including the dentate gyrus. NgR2 mRNA expression was also noted in thalamus, habenula and amygdala. Following kainic acid we found a striking increase of NgR2 mRNA in the granule cell layer of the dentate gyrus (Fig. 2g, p = 0.005), that peaked at 12 h, thus later than the decrease of NgR1 mRNA in the same region. The levels of expression in CA1 were very stable (Fig. 2h, p = 0.62) with little variability over time and animals. In marked contrast, the expression in CA3 was significantly altered by kainic acid injection in the medial part (Fig. 2j, p = 0.001) but not in the lateral part (Fig. 2i, p = 0.1) even though the changes appeared to be of a similar nature. Expression was also stable in sensory (Fig. 2k, p = 0.95) and retrosplenial cortex (Fig. 2l, p = 0.44), as well as in thalamus (Fig. 2m, p = 0.32) and amygdala (Fig. 2n, p = 0.15). In summary there was significant expression of NgR2 mRNA in large regions of the brain and while in most areas transcript levels were not affected by kainic acid, there were significant increases in two regions, the dentate nucleus and the medial CA3 area of the hippocampal pyramidal layer.

### NgR3 is Upregulated by Kainic Acid

**Figure 3:**
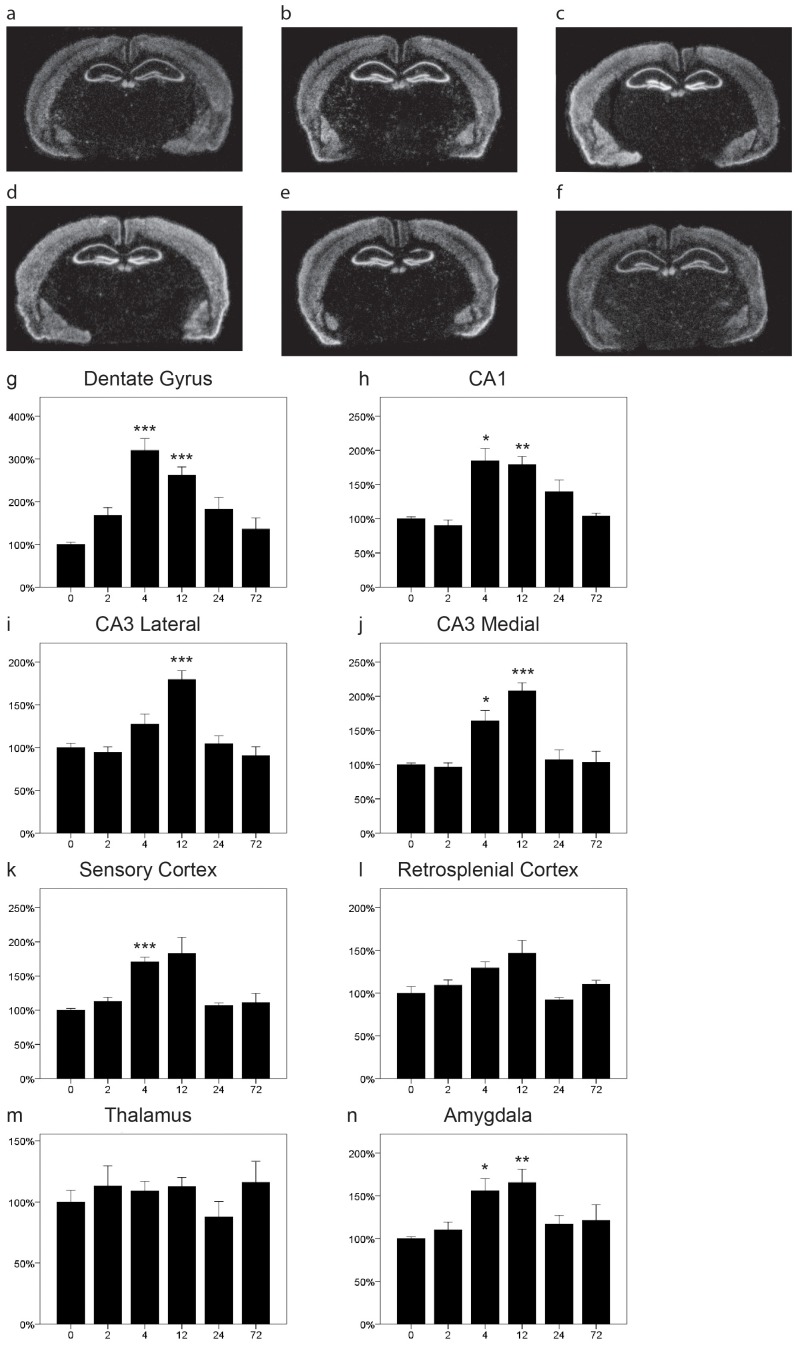
Distribution and regulation of NgR3 mRNA in the mouse brain. Representative coronal sections of control (a) and kainic acid injected (b 2 h, c 4 h, d 12 h, e 24 h, f 72 h) mice probed for NgR3 mRNA by quantitative in situ hybridization. In the dentate gyrus, NgR3 mRNA expression was significantly increased after 4 h, stayed significantly elevated for at least 12 h and then returned toward baseline levels (g). In CA1 (h) as well as in lateral (i) and medial (j) CA3 (h) expression of NgR3 mRNA significantly increased after kainic acid injection. NgR3 mRNA levels increased in both sensory (k) and retrosplenial cortex (l) as well as in amygdala (n) while thalamic levels were rather stable (m). Error bars represent S.E.M. and all expression values were normalized to expression in control mice (t = 0). *p<0.05, **p<0.01 compared to 0 h.

The transcriptional activity of NgR3, as reflected by the presence of NgR3 mRNA, had a distribution similar to that of NgR2 mRNA in the cerebral cortex, hippocampal CA1 and CA3 areas, the dentate gyrus, amygdala as well as habenula (Fig. 3). However, in contrast to NgR1 and 2, there was no or very low expression of NgR3 in thalamus. We also found that NgR3 transcript was specifically lacking (or very low) in CA2, which is not the case for NgR1 and NgR2 transcripts. NgR3 mRNA was strongly upregulated in CA1 (Fig. 3h, p<0.001) and in both the lateral (Fig. 3i p<0.001) and medial (Fig. 3j p<0.001) parts of CA3. There was a strong kainic acid induced increase of NgR3 transcript in the dentate gyrus (Fig. 3g, p<0.001) with a somewhat faster onset than the increases in CA1 and CA3. A significant upregulation was also observed in both sensory (Fig. 3k, p = 0.001) and retrosplenial cortex (Fig. 3l, p = 0.001). Thalamic expression of NgR3 was very low (or lacking), and did not appear to change with time (Fig. 3m, p = 0.664). NgR3 mRNA expression levels in amygdala increased in a pattern similar to that seen in CA3, with significant changes at 4 and 12 h (Fig. 3n, p = 0.005).

### LOTUS is Specifically Upregulated in the Dentate Gyrus and CA3 by Kainic Acid

**Figure 4:**
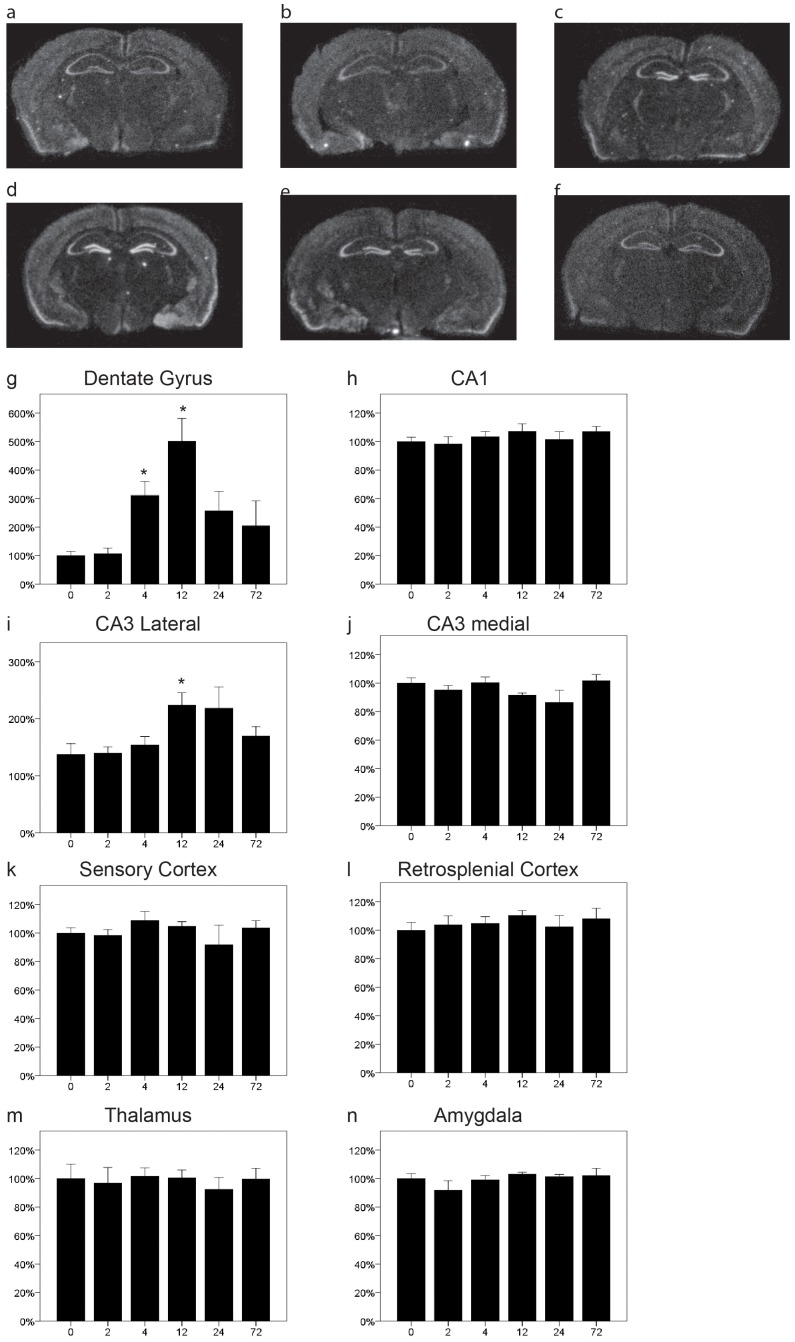
Distribution and regulation of Lotus mRNA in the mouse brain. Representative coronal sections of control (a) and kainic acid injected (b 2 h, c 4 h, d 12 h, e 24 h, f 72 h) mice probed for Lotus mRNA using quantitative in situ hybridization. There was a strong increase of LOTUS mRNA in the dentate gyrus following injection of kainic acid, reaching significance at 4 and 12 h (g). The expression of LOTUS mRNA after kainic acid was essentially unchanged in CA1 (h), while it increased in lateral (i) but not medial CA3 (j). The expression levels of LOTUS mRNA were stable in sensory (k) and retrosplenial cortex (l) as well as thalamus (m) and amygdala (n). Error bars represent S.E.M and all expression values were normalized to expression in control mice (t = 0). *p<0.05, **p<0.01 compared to 0 h.

In line with the notion of LOTUS as an NgR1 antagonist, we found that the regional expression pattern of LOTUS mRNA was rather similar to that of NgR1 mRNA, with LOTUS mRNA found in the cerebral cortex, hippocampus, amygdala and the dentate gyrus (Fig. 4). As LOTUS has been shown to be important for the formation of the lateral olfactory tract [29] it is also fitting that there was a marked expression in the olfactory associated area, the piriform cortex (Fig. 4). We found that the expression of LOTUS was not affected by kainic acid in most areas, CA1 (Fig. 4h, p = 0.67), amygdala, (Fig. 4n, p = 0.4), thalamus (Fig. 4m, p = 0.98), sensory (Fig. 4k, p = 0.71) and retrosplenial cortex (Fig. 4l, p = 0.8). In the dentate gyrus, however, there was a strong, significant increase of LOTUS mRNA in response to kainic acid (Fig. 4g, p = 0.002). This increase peaked at 12 h but was significant already after 4 hours and seemed not to have returned to baseline by 24 h. We also noted a significant increase in the lateral, curved area of CA3 (Fig. 4i, p = 0.009) but not in the medial part (Fig. 4j, p = 0.13). Thus the pattern of response to the kainic acid challenge shown by LOTUS is different from any of the NgR genes in being relatively specific to the dentate gyrus and lateral CA3.

### Nogo-A Undergoes Complex, Moderate Changes

**Figure 5:**
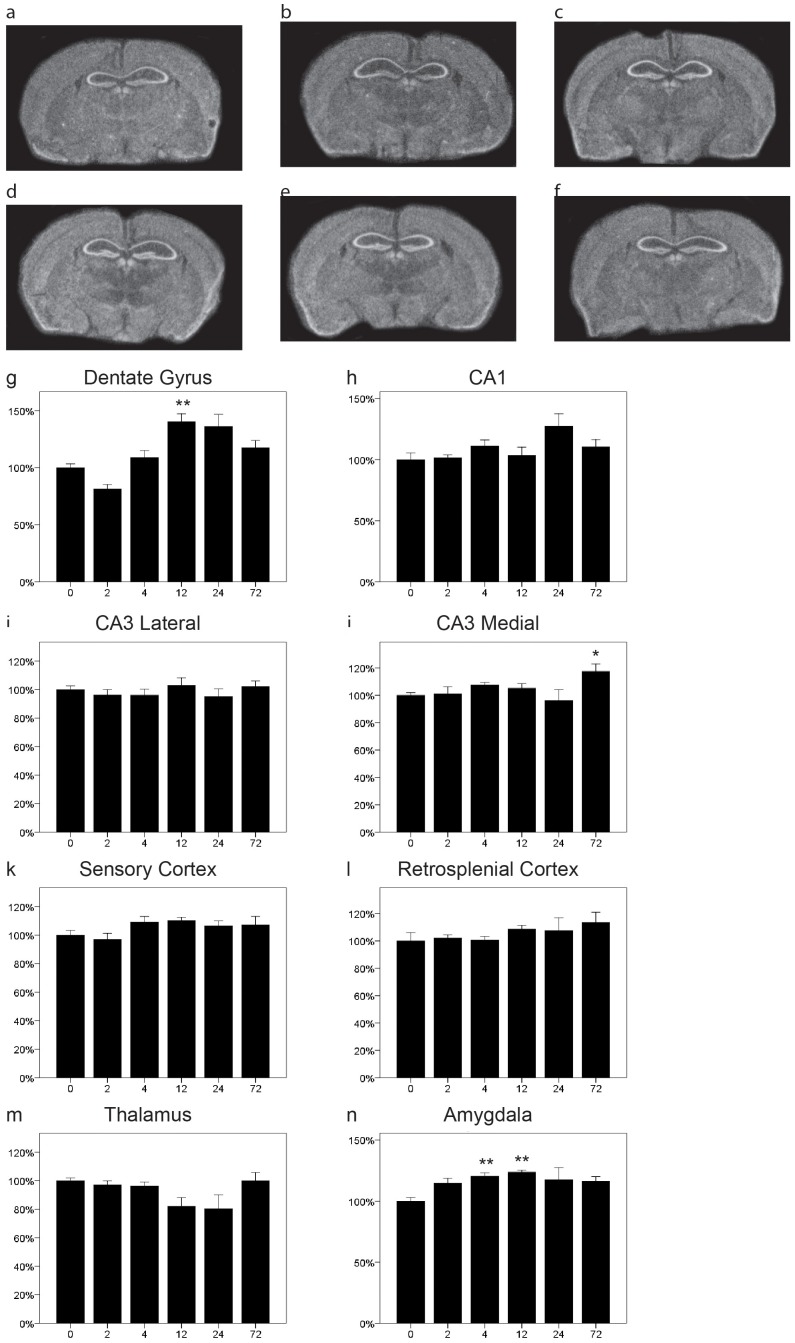
Distribution and regulation of Nogo-A mRNA in the mouse brain. Representative coronal sections of control (a) and kainic acid injected (b 2 h, c 4 h, d 12 h, e 24 h, f 72 h) mice probed for Lotus mRNA by quantitative in situ hybridization. The expression of Nogo-A mRNA was stable in most regions of the brain, including CA1 (h), lateral (i) and medial (j) CA3, sensory (k) and retrosplenial cortex (l) as well as thalamus (m). However, in the dentate gyrus (g) and the amygdala (n) expression of Nogo-A transcripts was significantly increased with a peak at 12 h (i). Error bars represent S.E.M and all expression values were normalized to expression in control mice (t = 0). *p<0.05, **p<0.01 compared to 0 h.

Nogo-A transcripts were found in most areas of the brain (Fig. 5), including both white and gray matter. We note quite strong Nogo-A transcript levels in hippocampus. Interestingly, we found a significant kainic acid-induced alteration of Nogo-A mRNA levels in the dentate gyrus (Fig. 5g, p<0.001), suggestive of an initial modest decrease (not significant), followed by an increase to supranormal levels that only slowly return towards control levels. In contrast, the expression in CA1 (Fig. 5h, p = 0.07), and lateral CA3 (Fig. 5i, p = 0.72) remained stable and were not markedly altered by Kainic acid. The medial part of CA3 did show a significantly increased expression of Nogo-A after 72 h but was stable at all other time points (Fig. 5j, p = 0.034). We also observed a significant increase of Nogo-A transcript levels in amygdala (Fig. 5n, p = 0.003) with a peak at 12 h, and which did not appear to return to baseline during the extent of the experiment. The expression in sensory (Fig. 5k, p = 0.121) and restrosplenial cortex (Fig. 5l, p = 0.34) did not change significantly, nor did the expression in thalamus (Fig. 5m, p = 0.17).

## Discussion

We have previously shown that NgR1 mRNA levels are downregulated by neuronal activation with the excitotoxic drug kainic acid, but also under more physiological conditions such as giving rats access to a running wheel [22]. Furthermore, area-specific downregulation of NgR1 mRNA occurs in those hind limb and neighboring forelimb somatosensory cortical areas that undergo functional plastic alterations following spinal cord injury in rats [21]. This suggests a link between down­regulation of NgR1 and increased plasticity in the adult cerebral cortex and fits well with McGee et al [24] showing that mice lacking NgR1 retain a high degree of ocular dominance shift plasticity into adulthood. We have also shown that even though mice that overexpress NgR1 in forebrain neurons have normal memory during the learning phase in Morris water maze and in passive avoidance tests, their ability to form lasting (>30 days) memories is severely impaired [25]. Hence temporary downregulation of NgR1 appears to be one key component of the presumably complex alterations that allows lasting memories to become embedded in the neuropil in the form of structural synaptic rearrangements.

To further address the role of neural activity-driven alteration of Nogo signaling, we have now investigated the possible regulation of the two NgR1 homologs NgR2 and NgR3, the endogenous NgR1 antagonist LOTUS, and the ligand Nogo-A. We confirm and extend our previous observations of NgR1 transcript changes in response to kainic acid and find that both NgR2 and NgR3 transcript levels are also regulated by kainic acid-induced neuronal activity. While NgR1 is downregulated in the dentate gyrus, both NgR2 and NgR3 are significantly upregulated following injections of kainic acid. For NgR3, this upregulation peaks at 4 h, which is similar to, or slightly later then the maximal downregulation of NgR1, while NgR2 reaches its peak somewhat after NgR1. In CA1, NgR1 mRNA levels are significantly downregulated while NgR3 is upregulated. Similarly NgR2 and NgR3 transcripts are also upregulated in CA3 while NgR1 is downregulated. We also find a significant upregulation of NgR3 in retrosplenial and sensory cortex. A possible explanation for the observed differential regulations of the different Nogo receptors could be to temporarily alter the efficacy of the different ligands. While NgR1 can bind to Nogo, OMgp, MAG and CSPGs, NgR2 has so far only been reported to bind MAG and NgR3 to bind to CSPG but none of the myelin derived growth inhibitors. The downregulation of NgR1 with concomitant upregulation of NgR2 and NgR3 would presumably increase local synaptic plasticity while nerve endings would remain sensitive to MAG and CSPGs, perhaps restricting plasticity to very local regions of the neuropil. The regulation that we found of NgR2 and NgR3 differs from that found by Wills et al. [23] who saw a decrease in cultured hippocampal neurons after increased neuronal activity. The reason for this discrepancy is not clear but could depend on the type of activation (KCl/NMDA vs kainic acid) or be due to a difference between their neuronal culture system and our in vivo analysis.

The levels of Nogo-A mRNA were stable in most regions of the brain but increased significantly in both the dentate gyrus and the amygdala after kainic acid injection. This increase in expression was temporally similar to that seen for NgR2 and NgR3 mRNA and occurs when NgR1mRNA has returned to baseline values. Hence, while NgR1 is rapidly downregulated within hours of the increased neuronal activity, several members of the Nogo-system will significantly increase their expression after 4–12 hours possibly to turn off the window of increased plasticity.

LOTUS has recently been identified as an endogenous NgR1 antagonist [29]. We find its distribution to be largely overlapping with that of NgR1, fitting with such a role. Interestingly, as demonstrated here, the specific upregulation of LOTUS mRNA levels in the dentate gyrus and in the lateral aspects of CA3 in response to strong neuronal activation by kainic acid would presumably assist in shutting down Nogo sensitivity in active neuronal circuits. The fact that robust upregulation of LOTUS mRNA was only found in these areas suggests a possible role for LOTUS in an early phase of memory formation, enhancing plasticity in the hippocampal formation. The upregulation of LOTUS transcripts follows the same temporal pattern as that of NgR2 and NgR3 transcripts in the dentate gyrus, but regulation differs substantially in other regions of the hippocampus (CA1, medial CA3). The reason for this is not clear but could indicate a more complicated control of Nogo-signaling in the dentate gyrus and lateral CA3.

It is well established that sensory inputs causes lasting alterations of synaptic structures [35]. Here we demonstrate that not only NgR1, but also NgR2, NgR3, Nogo-A and LOTUS are strongly regulated by increased neural activity, adding information to the presumably complex pattern of events needed to allow plasticity in the brain when lasting memories are to be formed, while maintaining structural integrity at other times. NgR1 remains unique by being the only one of these genes to respond to increased neuronal activity by downregulation of its transcriptional activity, while the other three investigated genes are upregulated. Together, the concerted regulations, with the downregulation of NgR1, the upregulation of Lotus, that could further decrease Nogo sensitivity of activated circuits, and NgR2 and NgR3 upregulation, possibly serving to restrict certain structural alterations, form a reasonable molecular machinery for controlled brain plasticity. While the present findings are only suggestive, their potential validity can be further tested by for instance genetic means in mice. A large number of other genes have been found to interact in various ways, directly or indirectly, during the process of forming lasting memories. The Nogo signaling system nevertheless stands out as a possible key downstream regulatory system in the important period of several days after a sensory input when the brain actively evaluates provisionally stored sensory experiences, leading to consolidation and permanent storage of the most important ones.
